# Exploring the molecular mechanisms underlying the potentiation of exogenous growth hormone on alcohol-induced fatty liver diseases in mice

**DOI:** 10.1186/1479-5876-8-120

**Published:** 2010-11-19

**Authors:** Ying Qin, Ya-ping Tian

**Affiliations:** 1 Department of Clinical Biochemistry, Chinese People's Liberation Army General Hospital, 28 Fu-Xing Road, Beijing 100853, PR China

## Abstract

**Background:**

Growth hormone (GH) is an essential regulator of intrahepatic lipid metabolism by activating multiple complex hepatic signaling cascades. Here, we examined whether chronic exogenous GH administration (via gene therapy) could ameliorate liver steatosis in animal models of alcoholic fatty liver disease (AFLD) and explored the underlying molecular mechanisms.

**Methods:**

Male C57BL/6J mice were fed either an alcohol or a control liquid diet with or without GH therapy for 6 weeks. Biochemical parameters, liver histology, oxidative stress markers, and serum high molecular weight (HMW) adiponectin were measured. Quantitative real-time PCR and western blotting were also conducted to determine the underlying molecular mechanism.

**Results:**

Serum HMW adiponectin levels were significantly higher in the GH1-treated control group than in the control group (3.98 ± 0.71 μg/mL vs. 3.07 ± 0.55 μg/mL; *P *< 0.001). GH1 therapy reversed HMW adiponectin levels to the normal levels in the alcohol-fed group. Alcohol feeding significantly reduced hepatic adipoR2 mRNA expression compared with that in the control group (0.71 ± 0.17 vs. 1.03 ± 0.19; *P *< 0.001), which was reversed by GH therapy. GH1 therapy also significantly increased the relative mRNA (1.98 ± 0.15 vs. 0.98 ± 0.15) and protein levels of SIRT1 (2.18 ± 0.37 vs. 0.99 ± 0.17) in the alcohol-fed group compared with those in the control group (both, *P *< 0.001). The alcohol diet decreased the phosphorylated and total protein levels of hepatic AMP-activated kinase-α (AMPKα) (phosphorylated protein: 0.40 ± 0.14 vs. 1.00 ± 0.12; total protein: 0.32 ± 0.12 vs. 1.00 ± 0.14; both, *P *< 0.001) and peroxisome proliferator activated receptor-α (PPARα) (phosphorylated protein: 0.30 ± 0.09 vs. 1.00 ± 0.09; total protein: 0.27 ± 0.10 vs. 1.00 ± 0.13; both, *P *< 0.001), which were restored by GH1 therapy. GH therapy also decreased the severity of fatty liver in alcohol-fed mice.

**Conclusions:**

GH therapy had positive effects on AFLD and may offer a promising approach to prevent or treat AFLD. These beneficial effects of GH on AFLD were achieved through the activation of the hepatic adiponectin-SIRT1-AMPK and PPARα-AMPK signaling systems.

## Background

Hepatic fat accumulation as a result of chronic alcohol consumption can induce liver injury. In the initial stage of alcohol-induced fatty liver disease (AFLD), triglycerides accumulate in hepatocytes inducing fatty liver (steatosis), although this process is reversible at this stage [1]. However, with continuing alcohol consumption, steatosis can progress to steatohepatitis, fibrosis, cirrhosis and even hepatocellular carcinoma [2]. Thus, it is crucial to develop specific pharmacological drugs to treat alcoholic steatosis during the early stage of AFLD and prevent the progression to more severe forms of liver damage.

There is growing evidence to suggest that the adiponectin-sirtuin 1 (SIRT1)-AMP-activated kinase (AMPK) signaling system is an essential regulator of hepatic fatty acid oxidation and is inhibited by chronic alcohol exposure. Furthermore, this pathway is closely associated with the pathogenesis of AFLD [3]. Adiponectin, an adipokine that is exclusively secreted by adipocytes, plays an important role in regulating systemic energy metabolism and insulin sensitivity *in vivo*. Adiponectin was also reported to be effective in alleviating alcohol- and obesity-induced hepatomegaly, steatosis and serum alanine transaminase (ALT) abnormalities in mice [4]. SIRT1 is a NAD^+^-dependent class III protein deacetylase that regulates lipid metabolism through deacetylation of modified lysine residues on histones and transcriptional regulators [5-7]. AMPK is a heterotrimeric protein consisting of one catalytic subunit (α) and two non-catalytic subunits (β and γ). Activated AMPK can phosphorylate its downstream substrates to act as a metabolic switch to regulate glucose and lipid metabolism [8-10]. Furthermore, activation of the adiponectin-SITR1-AMPK pathway increases the hepatic activities of peroxisome proliferator activated receptor-γ (PPARγ) and PPARα coactivator (PGC1), and decreases the activity of sterol regulatory element binding protein 1 (SREBP-1) in several animal models of AFLD [7,11-13]. PGC1 and SREBP-1 are the key transcriptional regulators of genes controlling lipogenesis and fatty acid oxidation [7,14-16].

Growth hormone (GH) is an important regulator of intrahepatic lipid metabolism. Hepatic GH can interact with its receptor (GHR) on the surface of target cells and induces the association of GHR with Janus kinase (JAK)-2 to initiate tyrosine phosphorylation of GHR and JAK2. Phosphorylation of GHR and JAK2 consequently activates multiple signaling cascades by phosphorylating a series of downstream signaling molecules, including p38 mitogen-activated protein kinase (p38-MAPK), AMPK and PPARα [18-20]. The activated signaling molecules regulate the transcription of GH-responsive genes in the liver. Inhibition of endogenous hepatic GH signaling might perturb lipid metabolism and induce liver steatosis [21]. Our previous study showed that exogenous GH can prevent non-alcoholic fatty liver disease (NAFLD). Cross-talk among GH regulative signaling pathways can inhibit lipid synthesis, reduce hepatic triglyceride (TG) accumulation, enhance glucose metabolism and inhibit gluconeogenesis in the liver, and can thus reverse hepatic steatosis and fibrosis [20].

Here, we explored the effects and molecular mechanisms of GH on AFLD. Viral vectors can induce longer-lasting effects than recombinant protein administration and thus avoid the inconvenience of repetitive subcutaneous injections. Therefore, we used GH gene delivery technology rather than recombinant GH injection in this study. The coding sequence (cds) for the GH1 gene (human GH [hGH]; GenBank accession number NM_000515) was transferred *in vivo *by recombinant adeno-associated viral vectors pseudotyped with viral capsids from serotype 1 (rAAV2/1), as previously described [22].

## Methods

### rAAV2/1 vector containing the GH1 gene

The method used to construct the rAAV2/1 vector containing the GH1 gene is described in more detail elsewhere [20,22]. In brief, GH1 was cloned from a PCR product using 5'-CA**GAATTC**GCCACCATGGCTACAGGCTCCCGG-3' (sense primer) and 5'-CTGC**GTCGAC**GAAGCCACAGCTGCCCTC-3' (antisense primer) (*EcoR*I and *Sal*I restriction sites are indicated in bold/underlined) from the template of a pUC19 plasmid DNA containing GH1 (Xinxiang Medical University, Xinxiang, Henan Province, China). The 677-bp GH1 DNA fragment (including the 651-bp cds) was digested with *Sal*I and *Eco*RI and inserted into the *Sal*I and *Eco*RI sites of the pSNAV2.0 vector (AGTCGene Technology Co. Ltd., Beijing, China). rAAV2/1 production and purification were performed as previously described [23]. The viral genome particle titer (1.0 × 10^12 ^v.g./mL) was determined by quantitative DNA dot-blots [24].

### Animal study

Male C57BL/6J mice weighing 25.0 ± 2.0 g were obtained from the Institute of Laboratory Animal Sciences, Chinese Academy of Medical Sciences & Peking Union Medical College (Beijing, China) and housed in stainless steel wire-bottomed cages with a 12-h light/dark cycle. Animal experiments were performed in accordance with the guidelines of the National Institutes of Health (Bethesda, MD, USA) and the Chinese People's Liberation Army General Hospital for the humane treatment of laboratory animals.

Mice were fed a liquid diet and distributed into six groups: control and GH1-treated control (control groups); alcohol and GH1-treated alcohol (alcohol groups); pair-fed I and pair-fed II (pair-fed groups). The diet was based on the Lieber-DeCarli formulation, and contained 35% of calories from fat (corn oil), 12% from carbohydrate, 18% from protein, and 35% from ethanol (alcohol groups) or isocaloric maltose dextrin (control and pair fed groups). The ethanol concentration was gradually increased from 17% to 35% during the first week of feeding and then maintained at the same concentration for another 5 weeks [25]. Food intake was recorded daily in the control and alcohol groups. The food intake in the pair-fed groups I and II was matched to the respective alcohol-fed groups. One week after alcohol administration, mice in the GH1-treated control and GH1-treated alcohol-fed groups were intravenously injected with a single dose of 1.0 × 10^11 ^rAAV2/1-CMV-GH1 viral particles into the tail vein. The survival study was repeated on three occasions to determine the survival rate (18 mice per group on each occasion for the alcohol group and GH1-treated alcohol group; 6 mice per group on each occasion for all of the other groups). The survival rate in each group was calculated as the number of survivors/total number of animals in each group × 100%.

Six weeks later, six of the surviving mice from each group were weighed and then euthanized, at which time blood, liver tissue, and adipose tissues were collected. The perirenal and epididymal fat pads were pooled (visceral fat, VF) and weighed using a precision electronic balance (AV264; Ohaus, Pine Brook, NJ, USA) to determine VF percentage (VF%) of total body weight (VF weight/body weight × 100%). The hepatic index (HI) was calculated as liver weight/body weight × 100%.

### Hepatic histology and measurement of triglyceride content

Fresh liver sections were fixed in 4% paraformaldehyde, dehydrated, embedded in paraffin, and sectioned. Formalin-fixed, paraffin-embedded sections were cut (5 μm thick) and mounted on glass slides. The sections were deparaffinized in xylene and stained with hematoxylin and eosin using standard techniques. Hepatic steatosis was classified into four grades based on fat accumulation using the method devised by Brunt et al [26]. Briefly, grade 0 indicates no fat in the liver, while grades 1 (light), 2 (mild) and 3 (severe) were defined as the presence of fat vacuoles in < 33%, 33-66% or > 66% of hepatocytes, respectively. The fat deposition pattern was classified as macrovesicular, microvesicular, or mixed. Biopsies were examined by two investigators blind to the treatment groups. The κ value was calculated to determine the inter-observer agreement. Hepatic TG levels were measured as previously described [27].

### Mouse serum assays

Insulin-like growth factor 1 (IGF-1; ADL, Alexandria, VA, USA), insulin (ADL) and tumor necrosis factor-α (TNFα; R&D Systems, Minneapolis, MN, USA) were measured using enzyme-linked immunosorbent assay kits. Serum ethanol levels (blood alcohol concentration, BAC) achieved in the mice after chronic ethanol administration for 6 weeks were measured using a blood alcohol test kit (Abbott laboratories, Abbott Park, IL, USA). Serum β-hydroxybutyrate (β-OHB) was measured using a colorimetric method (Stanbio, Boerne, TX, USA). Serum levels of glucose, alanine aminotransferase, TG and total cholesterol (TC) were determined using standard methods. Insulin resistance (IR) was assessed using the homeostasis model assessment of IR (HOMA-IR) as blood glucose × blood insulin/22.5 [28,29].

### Lipid peroxidation

Malondialdehyde (MDA) was quantified using the thiobarbituric acid reaction, as previously described [30,31], and measured using a thiobarbituric acid reactive substances assay (Cayman Chemical Co. Inc., Ann Arbor, MI, USA). In brief, 25 mg of liver tissue was added to 250 μl of radioimmunoprecipitation assay buffer containing protease inhibitors. The mixture was sonicated for 15 s at 40 V over ice and centrifuged at 1600 ×*g *for 10 min at 4°C. The supernatant was used for analysis.

### Real-time quantitative polymerase chain reaction (qRT-PCR)

Total RNA was extracted from liver and adipose tissue samples and isolated and purified with TRIzol reagent (Invitrogen, Carlsbad, CA, USA) and a NucleoSpin^® ^RNA clean-up kit (Macherey-Nagel, Duren, Germany). Fifty nanograms of total RNA were used in qPT-PCR reactions. qRT-PCR amplification was conducted in a LightCycler (Roche Diagnostics, Pleasanton, CA, USA) using a LightCycler-FastStart DNA Master SYBR Green I Kit (SuperArray Bioscience, Frederick, MD, USA). The following qRT-PCR primer sets were purchased from SuperArray Bioscience: SIRT1 (PPM05054A), GPAT1 (PPM33295A), FAS (PPM03816E), SCD1 (PPM05664E), ACCα (PPM05109E), ME (PPM 05495A), MCAD (PPM25604A), AOX (PPM04407A), CPT1a (PPM25930B), FOXO1 (PPM03381B), PGC1α (PPM03360E), adipoR1 (PPM35710A), adipoR2 (PPM 38032E), and PPARα (PPM 05108B). All samples and standards were amplified in triplicate. Target mRNA was calculated using the comparative cycle threshold (Ct) method by normalizing the target mRNA Ct for that of GAPDH.

### Western blotting and PGC1α acetylation assays

Liver nuclear protein or whole protein were extracted and used for western blotting which was performed as previously described [20]. Total AMPKα, phospho-AMPKα (p-AMPKα), phospho-ACC (p-ACC) and PGC1α were visualized using primary antibodies from Cell Signaling Technology (Danvers, MA, USA). SIRT1 and SREBP-1c were visualized using antibodies obtained from Santa Cruz (Santa Cruz, CA, USA). Nonspecific proteins were used as loading controls to normalize the signal obtained for liver nuclear protein extracts. N-acetyl-leucinal-leucinal-norleucinal (25 μg/mL) (Calbiochem, San Diego, CA, USA) was present in all procedures for nuclear SREBP-1c (nSREBP-1) analysis. Polyclonal rabbit anti-GAPDH antibody (Sigma-Aldrich Co., St. Louis, MO, USA) was used to normalize the signal obtained for total liver protein extracts. The working dilution for antibodies ranged from 1:500 to 1:2,000. PGC1α levels and acetylation were detected using specific antibodies for PGC1α and acetyl lysine, respectively (Cell Signaling Technology) [12,13].

### Statistical analyses

Western blots were quantified using Image-Pro Plus software version 6.0 (Media Cybernetics Incorporated, Silver Spring, MD, USA). Data are means ± standard deviation. Statistical analyses were done using SPSS software version 13.0 (SPSS Inc., Chicago, IL, USA). Student's t-test, one way-ANOVA, Kruskal-Wallis one-way ANOVA on ranks and two-way analysis of variance (followed by post hoc protected least square difference tests) was used for other statistical analysis. Values of *P *< 0.05 were considered significant.

## Results

### Survival rate

**Figure 1:**
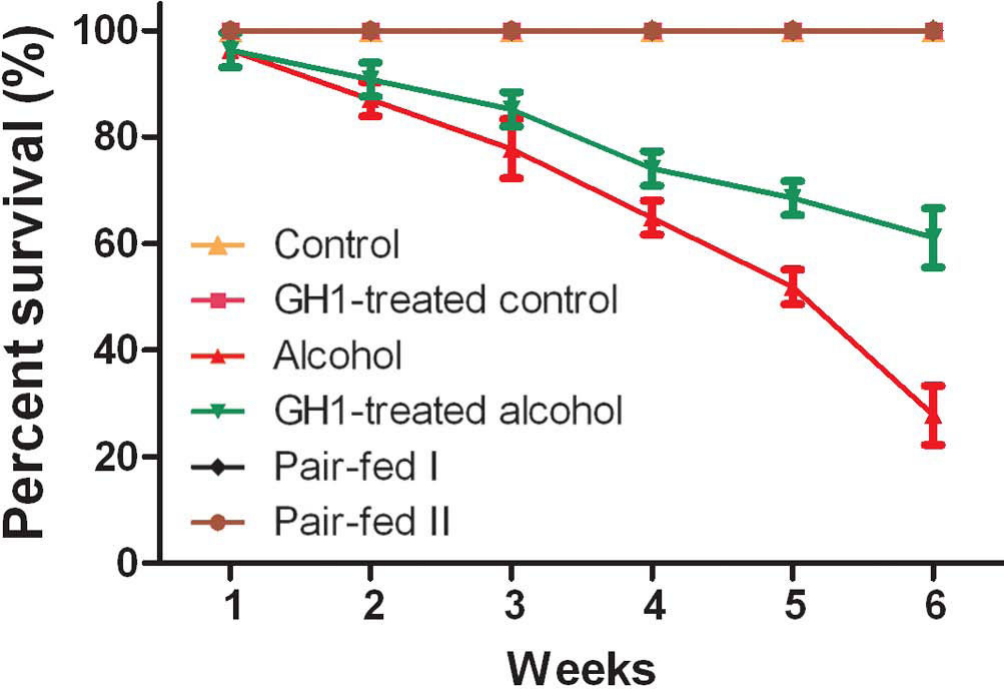
**Survival rates**. The survival rate was 100% at baseline and decreased to 24.07 ± 3.21% in the alcohol-fed group and 66.96 ± 5.56% in the GH1-treated alcohol-fed group after 6 weeks of treatment. The survival rate was maintained at 100% in the other groups. n = 18 mice per group for the alcohol and GH1-treated alcohol groups; n = 6 mice per group for the other groups.

Survival analysis showed that the mice in the alcohol-fed group began to die at the first week of alcohol administration, with a survival of 24.07 ± 3.21% after 6 weeks of alcohol administration (Figure 1). Chronic alcohol administration also decreased the activity of mice and induced immobility and grouping, and the growth of coarse hair. There are no obvious differences in survival rate between the alcohol and GH1-treated alcohol groups at the start of alcohol administration. However, GH1 treatment significantly slowed the decrease in survival rate at 3 weeks after starting alcohol administration (85.18 ± 3.21% vs. 77.78 ± 5.56%; *P *< 0.05). At the end of the experiments, the survival rate was 66.96 ± 5.56% in the GH1-treated group, about 2.8-fold higher than that in the untreated alcohol group (*P *< 0.001) (Figure 1). The reason for the delayed onset of GH effects on alcohol feeding may be that significant transgene expression following rAAVs-mediated gene transfer is not observed for 1-2 weeks, reaching a plateau by 4-6 weeks. The expression delay is primarily determined by the uncoating efficacy of vector genomes [32]. Nevertheless, GH administration increased the survival rate and improved the general health condition of the surviving mice at the end of experiment (Figure 1). Very few deaths occurred in the control, GH1-treated control, and pair-fed I and II groups (Figure 1), and mice in these groups remained healthy.

### GH1 gene expression in AFLD mice

We observed the development of the typical histological and biochemical features of liver steatosis in the AFLD mice models after 6 weeks of alcohol exposure. GH1 gene expression can be sustained for at least 6 months after a single injection of rAAV2/1-CMV-GH1, as we have reported elsewhere [22]. The alcohol diet did not cause marked changes in serum IGF-1 levels, which were similar to those in the control group (384.53 ± 38.75 ng/mL vs. 393.95 ± 46.65 ng/mL, *P *> 0.05). However, IGF-1 was slightly but not significantly higher in the GH1-treated control (415.32 ± 39.97 ng/mL) and GH1-treated alcohol-fed groups (400.55 ± 50.78 ng/mL) compared with the control group (*P *> 0.05, Table1). The serum insulin level in the alcohol-fed group was 24.47 ± 1.92 μU/mL, which was similar to that in the control group (24.90 ± 2.19 μU/mL; *P *> 0.05). The serum insulin levels in the GH1-treated control and GH1-treated alcohol-fed groups were 25.89 ± 2.45 μU/mL and 25.60 ± 2.43 μU/mL, respectively, which were slightly, but not significantly higher than that in the control group (*P *> 0.05). The changes in serum glucose levels showed similar trends to those of insulin. As a result, although GH1 treatment did not significantly elevate the serum levels of insulin and glucose, it did significantly increase HOMA-IR in the GH1-treated control group and GH1-treated alcohol group as compared with the control group (7.85 ± 0.61 vs. 7.14 ± 0.56 and 7.71 ± 0.38 vs. 7.14 ± 0.56, respectively; both *P *< 0.05, Table 1).

**Table 1:** Metabolic parameters

	Control	GH1-treated control	Alcohol	GH1-treated alcohol	Pair-fed I	Pair-fed II
BAC (mmol/l)	-	-	22.13 ± 2.28	16.20 ± 1.54^###^	-	-
β-OHB (μmol/l)	88.5 ± 10.48^d^	246.4 ± 41.2^a^	122.7 ± 15.36^c^	164.53 ± 19.80^b^	94.87 ± 10.43^d^	92.80 ± 11.98^d^
**TG (mmol/l)**	1.24 ± 0.31	1.13 ± 0.13	1.54 ± 0.22*	1.16 ± 0.28	1.15 ± 0.25	1.17 ± 0.32
**TC (mmol/l)**	4.04 ± 0.48	3.95 ± 0.36	3.91 ± 0.47	3.99 ± 0.60	4.10 ± 0.35	4.08 ± 0.28
**IGF-1 (ng/ml)**	393.95 ± 46.65	415.32 ± 39.97	384.53 ± 38.75	400.55 ± 50.78	399.05 ± 34.67	387.37 ± 21.68
Insulin (μU/ml)	24.90 ± 2.19	25.89 ± 2.45	24.47 ± 1.92	25.60 ± 2.43	24.60 ± 1.88	24.76 ± 2.42
**Glucose (mmol/l)**	6.46 ± 0.36	6.85 ± 0.45	6.23 ± 0.25	6.79 ± 0.42	6.32 ± 0.43	6.28 ± 0.47
**HOMA-IR**	7.14 ± 0.56	7.85 ± 0.61* ^##^	6.78 ± 0.62	7.71 ± 0.38* ^#^	6.92 ± 0.92	6.88 ± 0.47
TNFα (pg/ml)	8.50 ± 2.03^#^	7.95 ± 1.75^##^	10.83 ± 2.07*	8.77 ± 1. 94^#^	8.20 ± 1.77^#^	8.80 ± 1.93^#^
**Hepatic MDA **(μM)	3.39 ± 1.09	2.27 ± 0.67* ^##^	3.77 ± 1.03	2.87 ± 0.81	3.17 ± 0.92	3.05 ± 0.59

n = 6 mice per group. *P < 0.05 vs. the control group; ^#^*P *< 0.05, ^##^*P *< 0.01 or ^###^*P *< 0.001 vs. the alcohol group. HOMA-IR: homeostasis model assessment of insulin resistance; MDA: malondialdehyde; β-OHB: β-hydroxybutyrate; TC: total cholesterol; TG: triglyceride; TNFα: tumor necrosis factor-α.

### Food intake, body composition and HI

**Figure 2:**
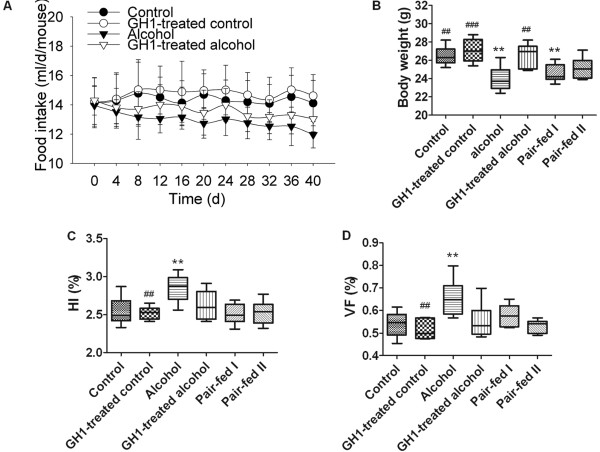
**Food intake and body composition**. **(A) **Daily food intake. **(B) **Body weight. **(C) **Hepatic index. **(D) **Visceral fat percentage. HI: hepatic index. VF%: visceral fat percentage. Error bars represent standard deviations. n = 6 mice per group. *P < 0.05 or **P < 0.01 vs. the control group; ^#^*P *< 0.05, ^##^*P *< 0.01 or ^###^*P *< 0.001 vs. the alcohol group.

GH1 administration had apparent effects on the appetite of alcohol-fed mice. The alcohol-fed group showed a slow and progressive reduction in mean food intake, decreasing to 12.0 ± 0.9 mL/d/mouse on day 40, compared with 14.1 ± 1.5 mL/d/mouse in the control group (*P *< 0.01). Appetite was partly reversed by GH1 treatment (13.0 ± 0.9 mL/d/mouse; *P *< 0.05 vs. the alcohol-fed group). The mean food intake was slightly but not significantly higher in the GH1-treated control group than in the control group throughout the experiment (Figure 2A).

The body weight in the control group was 26.47 ± 1.02 g at the end of the study but did not increase significantly versus baseline, and tended to decrease in the pair-fed II group (25.13 ± 1.17 g), but not significantly. The body weight of the alcohol-fed and pair-fed I groups were 23.95 ± 1.36 g and 24.55 ± 0.98 g, respectively, and was significantly lower than that in the control group (both, *P *< 0.01). GH administration reversed the loss of body weight in the alcohol-fed group (26.17 ± 1.30 g; *P *< 0.01 vs. the alcohol-fed group). Body weight was higher in the GH1-treated control group (27.07 ± 1.26 g) than in the control group, although this was not statistically significant (Figure 2B).

Both HI (2.85 ± 0.18% vs. 2.54 ± 0.19%, respectively; *P *< 0.01) and VF% (0.66 ± 0.08% vs. 0.54 ± 0.06%, respectively; *P *< 0.01) were significantly higher in the alcohol-fed group than in the control group, despite decreases in appetite and body weight in the alcohol-fed group compared with the control group. The HI and VF% were both reduced to control levels in the GH1-treated alcohol-fed group (2.69 ± 0.20% and 0.55 ± 0.08%, respectively; both, *P *> 0.05 vs. the control group; *P *< 0.05 and *P *< 0.01 vs. the alcohol-fed group). The decreases in food intake in the pair-fed groups did not cause obvious changes in HI (pair-fed I: 2.51 ± 0.13 g; pair-fed II: 2.53 ± 0.16; both, *P *> 0.05) or VF% (0.57 ± 0.05% and 0.53 ± 0.03%, respectively; *P *> 0.05), compared with the control group. These results suggest that exogenous GH improves body composition and prevents hepatomegaly in alcohol-fed mice, and thus ameliorated AFLD (Figure 2C, D).

### Liver steatosis in the AFLD mouse model

**Figure 3:**
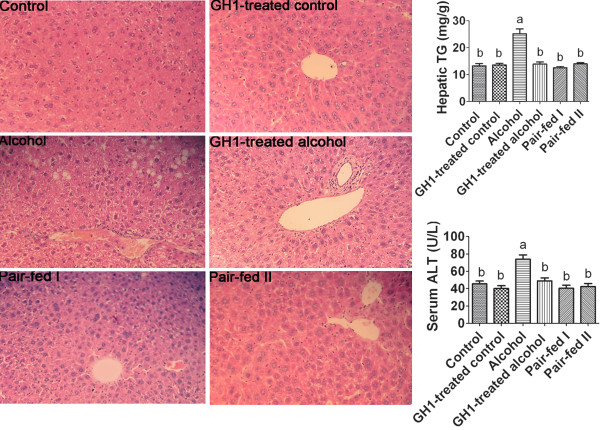
**Liver histology**. Accumulation of lipid droplets is evident in the liver of alcohol-fed mice, while relatively few lipid droplets were found in the hepatocytes of other groups (hematoxylin/eosin staining; original magnification, × 40). Serum ALT levels show similar trends to hepatic TG content in all groups. GH1 therapy reversed the alcohol-diet-induced increases in hepatic TG and serum ALT. ALT: alanine transaminase; TG: triglyceride. n = 6 mice per group. Means without a common letter differ at ***P ***< 0.05 vs. the control group.

The histological classification of steatosis in each group is summarized in Table 2. The inter-observer agreement was 0.83. The mean steatosis grade was lower in the GH1-treated alcohol-fed group (grade 1) than in the untreated alcohol-fed group (grade 2), which indicated that GH1 treatment prevented alcohol-induced accumulation of lipid droplets in the liver. Hepatic histologic and pathologic imaging revealed marked microvesicular or macrovesicular steatosis around the periportal zone, necrosis and inflammation, along with enlarged hepatocytes in the alcohol-fed mice (Figure 3). Notably, GH administration improved the steatosis condition in the alcohol-fed mice as there was much less hepatic accumulation of lipid droplets in these mice (Figure 3). Furthermore, fat deposition in the GH group was mainly microvesicular (Figure 3). Overall, hepatic steatosis was much less severe in the GH1-treated alcohol-fed mice than in the untreated alcohol-fed mice. Quantification of the hepatic lipid content was consistent with the histological findings. GH1 therapy alone did not affect the hepatic TG and serum ALT levels. The hepatic TG and serum ALT levels in the GH1-treated control group were 13.58 ± 1.48 mg/g and 40.10 ± 7.72 U/L, as compared with 13.23 ± 2.14 mg/g and 45.47 ± 7.96 U/L in the control group. However, alcohol feeding significantly increased hepatic TG and serum ALT levels to 25.17 ± 4.34 g and 73.85 ± 12.27 U/L, respectively, compared with the control group (both, *P *< 0.001; Figure 3), and these levels were restored to the normal levels by GH1 therapy to 13.88 ± 2.04 mg/g and 48.93 ± 8.12 U/L, respectively(both, *P *> 0.05 vs. the control group; both, *P *< 0.001 vs. the alcohol group) (Figure 3) In addition, the changes in serum TG and TNFα levels showed similar trends to that for hepatic TG and serum ALT (Table 1). By contrast, serum TC levels did not change markedly. Serum TC content was 4.04 ± 0.48 mmol/L in the control group, 3.95 ± 0.36 mmol/L in the GH1-treated control group, 3.91 ± 0.47 mmol/L in the alcohol group, and 3.99 ± 0.60 mmol/L in the GH1-treated alcohol group. Collectively, these results indicate that GH1 therapy seems to protect against further development of alcoholic liver steatosis in mice.

**Table 2:** Grading of hepatic steatosis.

Group	Steatosis grades			
	
	0	1	2	3
Control	6 (6)	0 (0)	0 (0)	0 (0)
GH1-treated control	6 (6)	0 (0)	0 (0)	0 (0)
Alcohol	0 (0)	3 (2)	2 (2)	1(2)
GH1-treated alcohol	0 (0)	6 (5)	0 (1)	0 (0)
Pair-fed I	6 (6)	0 (0)	0 (0)	0 (0)
Pair-fed II	6 (6)	0 (0)	0 (0)	0 (0)

n = 6 mice per group. κ value = 0.83, SE (*_k_*) = 0.083, *P *< 0.01

### Oxidative stress in the liver of AFLD mice

The hepatic MDA content (a lipid peroxidation product) was 3.39 ± 1.09 μM in the control group. Chronic alcohol administration induced modest oxidative stress although this was not significant, as evidenced by an increased hepatic MDA level (3.77 ± 1.03 μM; *P *> 0.05 vs. the control group) in the alcohol group. GH1 effectively reduced the hepatic MDA level in the control diet mice (2.27 ± 0.67 μM; *P *< 0.05 vs. the control group) and reduced the hepatic MDA levels in alcohol-fed mice to normal levels (2.87 ± 0.81 μM, *P *> 0.05 vs. both the control and alcohol-fed groups). The MDA levels in the pair-fed I and II groups were 3.17 ± 0.92 μM and 3.05 ± 0.59 μM, respectively, similar to that in the control group (Table 1).

### Exogenous GH upregulated adiponectin and increased hepatic adipoR2 expression in AFLD mice

**Figure 4:**
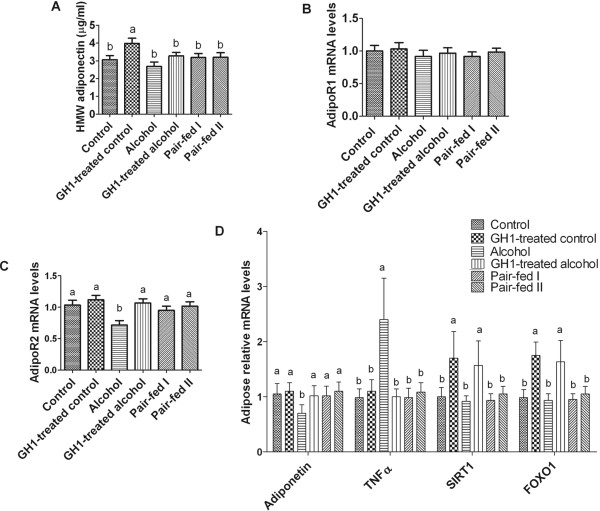
**GH1 therapy upregulated adiponectin and enhanced hepatic adipoR2 mRNA expression in alcohol-fed mice**. **(A) **Serum HMW adiponectin concentrations. **(B) **Relative mRNA levels of adipoR1. **(C) **Relative mRNA levels of adipoR2. **(D) **Relative adipose tissue mRNA levels of adiponectin, TNFα, SIRT1 and FOXO1. AdipoR1: adiponectin receptor 1; AdipoR2: adiponectin receptor 2; FOXO1: forkhead transcription factor O 1; HMW adiponectin: high molecular weight adiponectin; SIRT1, sirtuin 1; TNFα, tumor necrosis factor-α. n = 6 mice per group. Means without a common letter differ at ***P ***< 0.05 vs. control group.

Adiponectin plays a vital role in the prevention of alcoholic liver steatosis. Previous studies showed that GH regulates the expression of adiponectin and its receptors in adipocytes via the JAK2 and p38 MAPK pathways [4]. In our study, alcohol feeding lowered the serum HMW adiponectin levels in the alcohol group to 2.68 ± 0.62 μg/mL, although not significantly, compared with 3.07 ± 0.55 μg/mL in the control group (*P *> 0.05). GH1 therapy induced remarkable increases in serum HMW adiponectin concentrations in the control-fed group (3.98 ± 0.71 μg/mL; *P *< 0.001 vs. the control group), and reversed the HMW adiponectin level to normal levels in the alcohol-fed group (3.28 ± 0.49 μg/mL, *P *> 0.05 vs. the control and alcohol-fed groups) (Figure 4A). Alcohol feeding significantly reduced relative hepatic adipoR2 mRNA expression than that in the control group (0.71 ± 0.17 vs. 1.03 ± 0.19, respectively; *P *< 0.001), but did not inhibit hepatic adiponectin receptor 1 (adipoR1) mRNA expression (0.92 ± 0.23 vs. 1.00 ± 0.21, respectively; *P *> 0.05) (Figure 4B, C). GH1 therapy in the control diet group increased adipoR2 mRNA levels, although not significantly (Figure 4C). Moreover, GH1 therapy reversed the effect of alcohol feeding on adipoR2 by increasing the mRNA expression of adipoR2 to normal levels (1.07 ± 0.16; *P *> 0.05 vs. the control group; *P *< 0.001 vs. the alcohol-fed group,) (Figure 4B, C).

We also determined the mRNA expression of adiponectin, TNFα, SIRT1 and forkhead box transcription factor O 1 (FOXO1) in adipose tissues because adiponectin is expressed and secreted by adipose tissue. Figure 4D shows the relative expression levels of adiponectin and its possible regulators in adipose tissue. Alcohol feeding increased the relative TNFα mRNA expression compared with that in the control group (2.40 ± 0.75, *P *< 0.001 vs. the control group). Although alcohol feeding did not affect the mRNA expression of SIRT1 or FOXO1, GH1 therapy in alcohol-fed mice significantly increased the relative expression of SIRT1 (1.70 ± 0.48 vs. 1.00 ± 0.67, respectively; *P *< 0.001) and FOXO1 (1.76 ± 0.24 vs. 0.98 ± 0.15, respectively; *P *< 0.001), as compared with the control group. GH administration also suppressed TNFα expression and upregulated adiponectin gene expression to normal levels (the GH1-treated alcohol group vs. the alcohol group: TNFα, 1.00 ± 0.14 vs. 2.4 ± 0.75; adiponectin, 1.02 ± 0.18 vs. 0.70 ± 0.15; both, *P *< 0.001) (Figure 4D).

### Exogenous GH1 therapy stimulated hepatic AMPK and PPARα activity in AFLD mice

**Figure 5:**
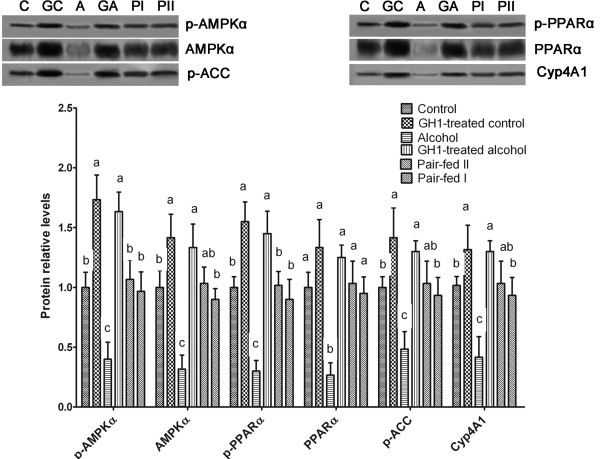
**GH1 therapy stimulated hepatic AMPK activity in alcohol-fed mice**. Western blotting of liver extracts was performed using anti-phosphorylated-AMP-activated protein kinase (AMPK)-α (anti-p-AMPKα), anti-AMPKα, anti-p- peroxisome proliferator activated receptor-α (PPARα), anti-PPAR-α, anti-phosphorylated acetyl CoA carboxylase (p-ACC) and anti-microsomal cytochrome P450, family 4, subfamily a, polypeptide 1 (Cyp4A1) antibodies. C: control group; GC: GH1-treated control group; A: alcohol group; GA: GH1-treated alcohol group; PI: pair-fed group I; PII: pair-fed group II. n = 6 mice per group. Means without a common letter differ at *P *< 0.05 vs. the control group.

Alcohol feeding significantly decreased the relative phosphorylated levels of hepatic AMPKα (0.40 ± 0.14 vs. 1.00 ± 0.12, respectively; *P *< 0.001) and PPARα (0.30 ± 0.09 vs. 1.00 ± 0.09, respectively; *P *< 0.001) compared with that in the control group, with a simultaneous decrease in the total protein levels of AMPKα (0.32 ± 0.12 vs. 1.00 ± 0.14, respectively; *P *< 0.001) and PPARα (0.27 ± 0.10 vs. 1.00 ± 0.13, respectively; *P *< 0.001) relative to the control group (Figure 5). Exogenous GH1 therapy restored the phosphorylated and total protein levels of AMPKα and PPARα in the livers of alcohol-fed mice (*P *< 0.001 vs. the alcohol-fed group; *P *> 0.05 vs. the control group) and therefore activated hepatic AMPK and PPARα in AFLD mice (Figure 5).

GH1-mediated activation of AMPK was accompanied by increased phosphorylation of acetyl-CoA carboxylase (ACC), a downstream target of AMPK, in the GH1-treated control (1.42 ± 0.25; *P *< 0.001 vs. the control group) and GH1-treated alcohol-fed (1.30 ± 0.09; *P *< 0.001 vs. the control group) groups (Figure 5). Its expression was suppressed in the alcohol-fed group (0.48 ± 0.15; *P *< 0.001 vs. the control group) and was restored by GH1-therapy in the GH1-treated alcohol group (*P *< 0.001 vs. both the control and alcohol-fed groups). The relative protein expression of hepatic microsomal cytochrome P450, family 4, subfamily A, polypeptide 1 (Cyp4A1) was significantly increased by GH1 therapy in the control (1.18 ± 0.50 vs. 1.00 ± 0.13; *P *< 0.001) and alcohol-fed (1.27 ± 0.15 vs. 1.00 ± 0.13; *P *< 0.05) groups, as compared with the control group (Figure 5). Its expression was also suppressed by the alcohol-diet, showing similar trends to those of ACC. Cyp4A1, a downstream target of PPARα, was assessed as a marker of PPARα activation *in vivo *[33]. These results indicate that exogenous GH1 therapy restores hepatic AMPK and PPARα activities, which were suppressed by alcohol feeding in mice.

### Exogenous GH1 therapy upregulated hepatic SIRT1 expression in AFLD mice

**Figure 6:**
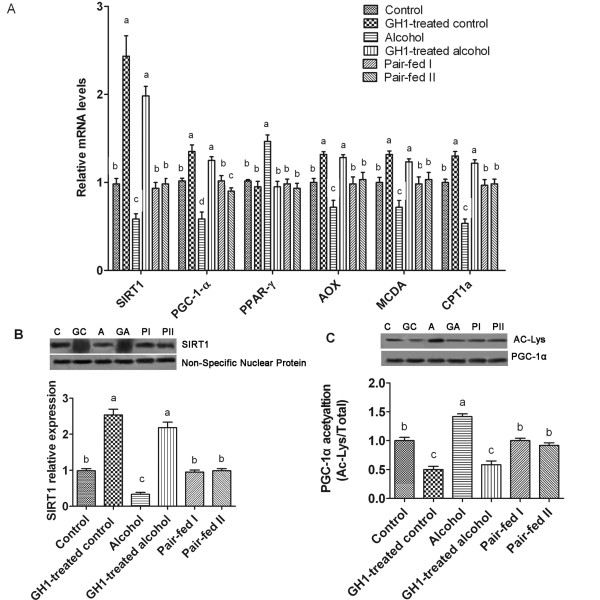
**GH administration upregulated hepatic SIRT1 expression in alcohol-fed mice**. **(A) **Hepatic mRNA expression of SIRT1. **(B) **SIRT1 protein levels. **(C) **Relative PGC1α acetylation. Hepatic nuclear SIRT1 protein levels were determined using an anti-SIRT1 antibody. A nonspecific nuclear protein band was used to confirm equal loading and to normalize the data. PGC1α was immunoprecipitated from liver extracts and immunoblotted with either an anti-acetylated lysine (Ac-Lys) antibody to determine the extent of PGC1α acetylation or with an anti-PGC1α antibody to determine the total amount of PGC1α. C: control group; GC: GH1-treated control group; A: alcohol group; GA: GH1-treated alcohol group; PI: pair-fed group I; PII: pair-fed group II. AOX: acyl-CoA oxidase; CPT1a: carnitine palmitoyltransferase 1a; MCDA: medium chain acyl-Co-A dehydrogenase; PGC1α: PPARα coactivator; PPARγ: peroxisome proliferator activated receptor-γ; SIRT1: sirtuin 1. n = 6 mice per group. Means without a common letter differ at *P *< 0.05.

Alcohol feeding reduced the relative mRNA (0.58 ± 0.15 vs. 0.98 ± 0.15; *P *< 0.05) and protein levels of hepatic SIRT1 (0.33 ± 0.12 vs. 0.99 ± 0.17; *P *< 0.01) compared with those in the control group. GH1 therapy significantly increased the relative mRNA (1.98 ± 0.15 vs. 0.98 ± 0.15; *P *< 0.001) and protein levels of SIRT1 (2.18 ± 0.37 vs. 0.99 ± 0.17; *P *< 0.001) in the alcohol-fed mice compared with the control group and the alcohol-fed group (Figure 6A, B). PPARγ, which may be regulated by SIRT1, is thought to be involved in the development of alcoholic and nonalcoholic fatty liver [14-16]. We found that chronic alcohol feeding increased the relative mRNA levels of PPARγ, as compared with that in the control group (1.47 ± 0.37 vs. 1.02 ± 0.04, respectively; *P *< 0.001) and this was reversed in the GH1-treated alcohol group (0.95 ± 0.15; *P *> 0.05 vs. the control group; *P *< 0.001 vs. the alcohol-fed group).

PGC1α is a marker of SIRT1 and AMPK levels and activities [7]. In the present study, alcohol feeding significantly reduced the relative PGC1α mRNA levels (0.58 ± 0.19 vs. 1.02 ± 0.08; *P *< 0.001) and significantly increased PGC1α acetylation (1.42 ± 0.12 vs. 1.00 ± 0.14; *P *< 0.001) compared with those in the control group (Figure 6C). GH1 treatment increased the relative mRNA expression of PGC1α (GH1-treated control group: 1.35 ± 0.19; GH1-treated alcohol: 1.25 ± 0.11; both, *P *< 0.001 vs. the control group; both, *P *< 0.001 vs. the alcohol-fed group) and decreased the extent of PGC1α acetylation (0.50 ± 0.14 and 0.58 ± 0.16, respectively; both, *P *< 0.001 vs. the control group; both, *P *< 0.001 vs. the alcohol-fed group). This suggests that the effects of GH1 administration are mediated by hepatic SIRT1 in AFLD mice (Figure 6A-C). In addition, GH1 therapy reversed the suppressive effects of alcohol on the gene expression of PGC1α-regulated fatty acid oxidation enzymes such as acyl-CoA oxidase (AOX), carnitine palmitoyltransferase 1a (CPT1a) and medium chain acyl-Co-A dehydrogenase (MCDA) (Figure 6A).

GH administration significantly reduced the BAC level in the GH1-treated alcohol group as compared with the alcohol-fed group (16.20 ± 1.54 mmol/l vs. 22.13 ± 2.28 mmol/l; *P *< 0.001). BAC was not detected in the other groups of mice (Table 1). Alcohol feeding modestly, but significantly increased the serum β-OHB levels versus that in the control group (122.70 ± 15.36 μmol/L vs. 88.50 ± 10.48 μmol/L; *P *< 0.01), which suggests that some of the hepatic free fatty acids in alcohol-fed mice might be converted to ketone bodies (Table 1). GH1 therapy dramatically increased the serum β-OHB levels in the control-fed (246.40 ± 41.2 μmol/L) and in the alcohol-fed groups (164.53 ± 19.80 μmol/L), as compared with that in the control or the alcohol-fed mice (all, *P *< 0.001). This suggests that GH administration upregulates hepatic fatty acid oxidation, promotes the generation of ketone bodies, and prevents alcohol-induced liver steatosis.

### Hepatic activity of the lipogenic transcription factor nSREBP-1c in alcohol-fed mice

**Figure 7:**
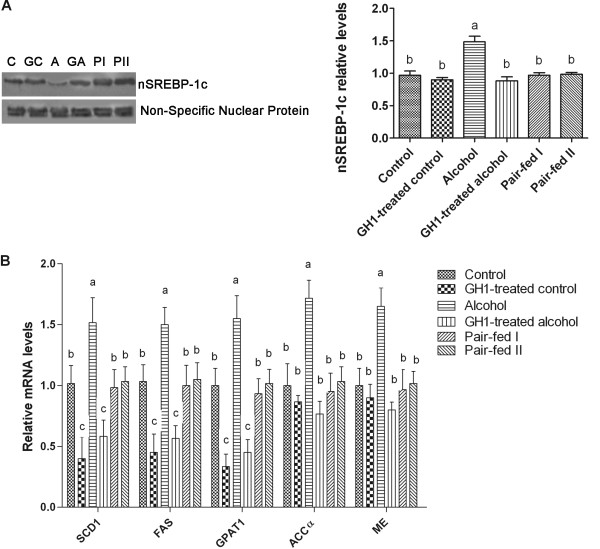
**GH1 therapy suppressed SREBP-1c activity and reduced the mRNA levels of SREBP-1-regulated genes encoding lipogenic enzymes in the livers of alcohol-fed mice**. **(A) **Nuclear SREBP-1c protein levels. **(B) **Relative mRNA levels of hepatic SREBP-regulated lipogenic enzymes. A nonspecific nuclear protein band in nuclear extracts was used to confirm equal loading and to normalize the data. C: control group; GC: GH1-treated control group; A: alcohol group; GA: GH1-treated alcohol group; PI: pair-fed group I; PII: pair-fed group II. ACCα: acetyl-CoA carboxylase-α; FAS: fatty acid synthase; GPAT1: glycerol-3-phosphate acyltransferase; ME: malic enzyme; nSREBP-1: nuclear sterol regulatory element binding protein 1; SCD1: stearoyl coenzyme A desaturase 1; SREBP-1, sterol regulatory element binding protein 1. n = 6 mice per group. Means without a common letter differ at *P *< 0.05.

SREBP-1c is regulated by SIRT1 as well as by AMPK [11-13]. In the present study, the relative protein expression of nSREBP-1c was significantly increased by chronic alcohol feeding, as compared with that in the control group (1.48 ± 0.21 vs. 0.96 ± 0.16; *P *< 0.001). GH1 therapy dramatically reduced hepatic nSREBP-1c protein expression in alcohol-fed mice to the normal levels (0.88 ± 0.15; *P *> 0.05 vs. the control group; *P *< 0.001 vs. the alcohol-fed group). However, the levels of nSREBP-1c in the GH1-treated control group were almost unchanged compared with those in the control group (0.90 ± 0.09; *P *> 0.05 vs. the control group) (Figure 7A). Chronic alcohol feeding increased the relative mRNA expression of several SREBP-1c-regulated lipogenic enzymes, including mitochondrial glycerol-3-phosphate acyltransferase (GPAT1) (1.55 ± 0.19 vs. 1.00 ± 0.14; *P *< 0.001), stearoyl coenzyme A desaturase 1 (SCD1) (1.52 ± 0.20 vs. 1.02 ± 0.15; *P *< 0.001), malic enzyme (ME) (1.65 ± 0.15 vs. 1.00 ± 0.14; *P *< 0.001), fatty acid synthase (FAS) (1.50 ± 0.14 vs. 1.03 ± 0.14; *P *< 0.001) and ACCα (1.72 ± 1.15 vs. 1.00 ± 0.18; *P *< 0.001) as compared with that in the control group (Figure 7B). GH1 therapy in the control diet-fed mice decreased the relative mRNA levels of these enzymes (GPAT1: 0.45 ± 0.10, *P *< 0.001; SCD1: 0.58 ± 0.13, *P *< 0.001; ME: 0.80 ± 0.06, *P *> 0.05; FAS: 0.57 ± 0.10, *P *< 0.001; ACCα: 0.76 ± 0.10, *P *> 0.05) relative to the control group, and induced equal or even greater decreases in the alcohol-fed group (GPAT1: 0.33 ± 0.10, *P *< 0.001; SCD1: 0.40 ± 0.18, *P *< 0.001; ME: 0.90 ± 0.11, *P *> 0.05; FAS: 0.45 ± 0.15, *P *< 0.001; ACCα: 0.87 ± 0.05, *P *> 0.05). These findings suggest that GH inhibits hepatic SREBP-1c activity in AFLD mice.

## Discussion

In this study, we examined whether chronic exogenous GH administration (via gene therapy) could improve alcoholic liver steatosis in mice, and we explored the underlying mechanisms. The animal model of AFLD was successfully established using a previously described method [7,25]. Chronic GH1 gene expression *in vivo *was achieved by a single injection of rAAV2/1-CMV**-**GH1, as we have described previously [20,22]. As would be expected, serum IGF-1 was also increased by GH1 therapy. We found that GH had positive effects in the AFLD mice by improving body composition, ameliorating serum lipid profiles, suppressing hepatocyte lipid droplet accumulation and decreasing oxidative stress.

GH is an essential regulator of intrahepatic lipid metabolism and can regulate many important signaling molecules to coordinate multiple lipid metabolism signaling pathways [4,22,34,35]. For example, GH can activate AMPK and PPARα in NAFLD rats [36,37]. Our recent study showed that GH administration has preventive effects against hepatic steatosis and fatty liver by regulating downstream genes through the phosphorylation or dephosphorylation of a group of signal transducers and activators in several hepatic signal transduction pathways [22].

Adiponectin can effectively alleviate hepatic steatosis in both AFLD and NAFLD [4,20]. It is rather interesting that GH can regulate adipocyte adiponectin and adipoR2 expression via the JAK2 and p38 MAPK pathways, and raise serum HMW adiponectin, the most active adiponectin isoform in the regulation of insulin and blood glucose levels [4,38]. Furthermore, GH regulates p85 expression and phosphoinositide-3-kinase activity in white adipose tissue while excess GH can induce insulin resistance. Upregulation of adipocyte adiponectin and adipoR2 by exogenous GH sensitizes adipocytes to the effects of adiponectin on insulin sensitivity by activating AMPK to stimulate glucose utilization and fatty acid oxidation. These activities may partially compensate and overcome exogenous GH-induced insulin resistance *in vivo *[39-42].

It is generally accepted that the adiponectin-SIRT1-AMPK signaling system plays a vital role in the development of AFLD [3]. Here, we showed that chronic exogenous GH therapy upregulated adiponectin and SIRT1 expression, and stimulated AMPK activity in the livers of chronically alcohol-fed mice. GH-mediated activation of the adiponectin-SIRT1-AMPK system was accompanied by increased circulating HMW adiponectin levels and enhanced hepatic adipoR2 mRNA expression. HMW adiponectin is the major bioactive isoform of adiponectin, and is responsible for the insulin-sensitizing effects of adiponectin, while adipoR2 is the predominant adiponectin receptor in the liver [36]. Since SIRT1 can positively regulate FOXO1 activity [37,43], the hepatic SIRT1-FOXO1 axis may also be involved in adipoR2 mRNA upregulation. Moreover, recent studies have shown that activated SIRT1 could act upstream of AMPK by modulating LKB1--an upstream AMPK kinase--which may serve as a key component in the lipid-lowering effect in hepatic cells and in the liver *in vivo *[7,44]. Hence, we deduced that the protective effects of exogenous GH against alcohol-induced liver steatosis may be realized, at least in part, by turning on the hepatic adiponectin-SIRT1-AMPK signaling system and related signaling pathways to ameliorate alcohol-induced impairments in the signaling pathways controlling lipid metabolism.

Furthermore, GH therapy increased PGC1α activity and restored the mRNA levels of several PGC1α target genes encoding fatty acid oxidation enzymes in chronic alcohol-fed mice. We also found that GH therapy reduced hepatic SREBP-1c protein levels and decreased the mRNA expression of SREBP-1c target genes encoding lipogenic enzymes in alcohol-fed mice. These findings also indicate that GH administration coordinates the adiponectin-SIRT1-AMPK signaling system to modulate its downstream signaling molecules that regulate the transcription of alcohol-responsive genes. These pathways ultimately upregulated fatty acid oxidation, reduced lipid synthesis, and prevented hepatic lipid accumulation in alcohol-fed mice.

The hepatic targets of GH include the JAK2/STAT3 (STAT5), p38 MAPK, AMPK, ERK1/2 and PPARα signaling pathways. We recently reported that GH administration can activate AMPK-PPARα signaling [25]. PPARα is centrally involved in the regulation of lipid homeostasis and is essential for normal liver function. PPARα mainly participates in fatty acid β-oxidation and plays an important role in modulating hepatic TG accumulation. Inhibition of PPARα signaling may impair lipoprotein transport, reduce fatty acid oxidation and enhance lipogenesis, which ultimately induces the development of steatosis [35,45]. In this study, we found that, alcohol downregulated hepatic expression of PPARα and CYP4A1, a typical downstream target of PPARα. Meanwhile chronic exogenous GH restored PPARα and CYP4A1 expression, which probably contributed to the improvements in alcoholic fatty liver. These results suggest that other pathways, in addition to the adiponectin-SIRT1-AMPK system, may improve AFLD in response to GH therapy.

An earlier study revealed that GH controls triglyceride synthesis and secretion by stimulating the expression of enzymes involved in *de novo *fatty acid and triglyceride synthesis. GH-induced stimulation of triglyceride secretion also seems to be linked to the degree of lipogenesis in the liver [46-48]. We found that GH administration increased the mRNA expression of the lipogenic enzymes ACC-1, FAS, SCD and GPAT, and their regulator SREBP-1c. These findings suggest that GH itself may directly improve AFLD by targeting the key transcriptional regulators of lipogenesis and fatty acid oxidation, in addition to the adiponectin-SIRT1-AMPK and AMPK-PPARα signaling pathways.

Although GH administration had positive effects on AFLD in terms of prevention and treatment, there are several limitations to be discussed. First, the molecular mechanisms underlying the effects of GH on the development of AFLD in the presence of alcohol are complex. Both alcohol and GH exert a myriad of effects *in vivo*, and it is unclear whether the protective effects of GH against AFLD are mediated directly or indirectly through the activation of multiple signaling cascades. Moreover, it is possible that GH influences signaling pathways other than those described in this study. Second, gene expression *in vivo *in response to exogenous GH may be confounded by the duration and dose of GH treatment. In addition, the abuse of GH by healthy subjects seeking its anabolic or lipolytic effects may impair glucose metabolism and increase insulin levels, and therefore enhance the oxidation of lipid substrates and result in insulin resistance. Furthermore, the safety and potential toxicity of GH gene therapy should not be neglected. Therefore, although this study offers a good starting point for the development of GH gene therapy for early prevention and treatment of AFLD, more studies are still needed.

## Conclusions

The present study suggests that GH administration can ameliorate AFLD by activating multiple hepatic signaling cascades, including the hepatic SIRT1-AMPK and PPARα-AMPK signaling pathways. GH may offer a novel and promising therapeutic target to treat ALFD in humans.

## Abbreviations

ACC: acetyl-CoA carboxylase; ACO: acyl-CoA oxidase; AdipoR1, adiponectin receptor 1; AdipoR2, adiponectin receptor 2; AFLD: alcoholic fatty liver disease; ALT: alanine transaminase; AMPK: AMP-activated protein kinase; AOX: acyl-CoA oxidase; BAC: blood ethanol concentration; β-OHB: β-hydroxybutyrate; CPT1a: carnitine palmitoyltransferase 1a; Cyp4A1: microsomal cytochrome P450, family 4, subfamily a, polypeptide 1; FAS: fatty acid synthase; FOXO1: forkhead transcription factor O 1; GH: growth hormone; GPAT1: glycerol-3-phosphate acyltransferase; HMW: adiponectin, high molecular weight adiponectin; HOMA-IR: the homeostasis model assessment of IR; IGF-1: insulin-like growth factor 1; IR: insulin resistance; JAK2: Janus kinase 2; MCDA: medium chain acyl-Co-A dehydrogenase; MDA: malondialdehyde; ME: malic enzyme; MTP: microsomal triglyceride transfer protein; NAFLD: non-alcoholic fatty liver disease; p38 MAPK: p38 mitogen-activated protein kinase; PGC-1: peroxisome proliferator activated receptor (PPAR)-γ and PPAR-α coactivator; PPARα: peroxisome proliferator activated receptor-α; Raav: recombinant adeno-associated virus; rAAV2/1, recombinant adeno-associated viral vectors pseudotyped with viral capsids from serotype 1; SCD1: stearoylcoenzyme A desaturase 1; SIRT1: sirtuin 1; SREBP-1: sterol regulatory element binding protein 1; STAT3: signal transducer and activator of transcription 3; STAT5: signal transducer and activator of transcription 5; TC: total cholesterol; TG: triglyceride; TNFα: tumour necrosis factor-α.

## Competing interests

The authors declare that they have no competing interests.

## Authors' contributions

Guarantor of integrity of entire study, Y.Q., and Y.P.T.; study concepts and design: Y. Q., and Y.P.T.; data acquisition/analysis/interpretation: Y. Q. and Y.P. T., statistical analysis: Y. Q.; obtained funding: Y. Q., and Y.P.T.; manuscript drafting or revision for important intellectual content, literature research, manuscript editing, and manuscript final version approval: Y. Q., and Y.P.T.
